# Clinical observation of mineralized collagen bone grafting after curettage of benign bone tumors

**DOI:** 10.1093/rb/rbaa031

**Published:** 2020-12-15

**Authors:** Chong Gao, Zhi-Ye Qiu, Jian-Wen Hou, Wei Tian, Jian-Ming Kou, Xi Wang

**Affiliations:** 1 Department of Orthopedics, The Second People’s Hospital of Lianyungang, No. 41 Hailiandong Road, Xinpu District, Lianyungang, Jiangsu 222006, China; 2 School of Materials Science and Engineering, Tsinghua University, Haidian District, Beijing 100085, China; 3 R&D Department, Allgens Medical Science Corporation, No. 26 Yongwangxi Road, Daxing District, Beijing 102629, China

**Keywords:** benign bone tumor, mineralized collagen, autogenous bone, bone grafting

## Abstract

Curettage of benign bone tumor is a common cause for bone defect. For such bone defect repair, autogenous bone, allogeneic bone and traditional artificial bone graft substitutes have many disadvantages. In recent years, a biomimetic mineralized collagen (MC) with similar composition and microstructures to the natural bone matrix was developed and used for treating various bone defects. In this work, a retrospective study analyzed clinical outcomes of patients treated with curettage of benign bone tumors and bone grafting with MC, in comparison to another group treated with the same surgical method and autogenous bone. Lane–Sandhu X-ray score of the autogenous bone group was superior to the MC group at 1 month after the operation, but the two groups had no statistical difference at 6 and 12 months. The MC group was better in Musculoskeletal Tumor Society scoring at 1 and 6 months after the operation, and the two groups had no statistical difference at 12 month. Therefore, the MC performed not as good as autogenous bone in early stage of bone healing but achieved comparable outcomes in long-term follow-ups. Moreover, the MC has advantages in function recovery and avoided potential complications induced by harvesting autogenous bone.

## Introduction

Bone defect caused by various reasons is very common in clinic. The curettage of benign bone tumors is one of the reasons resulting in bone defect [1]. For the treatment of bone defect, autogenous bone has been being considered as the gold standard because of its osteoconductivity, osteoinductivity and osteogenic cells contained in the bone tissue, but it gives rise to the complications of donor site, operative time, bleeding and so on [2, 3]. Moreover, the supply of autogenous bone is limited, especially for those usually with large bone defect after the removal of bone tumor [4–8]. In view of the above disadvantages, efforts have been made to find a new type of bone substitutes. Allogeneic bone grafts possess high risk of immune rejection and infection. Traditional sintered artificial bioceramics or acrylic bone cements are not degradable, they can be served as fillers for bone defects, and bioceramics with connective porous structure are able to conduct certain ingrowth of new bone tissues within the pores of the implants. For the bone defect after surgical treatment of benign bone tumor, the above-mentioned shortages by using allografts or traditional bone graft substitutes still exist. There is also a high recurrence risk of benign bone tumor after the curettage with any type of bone grafting, even autogenous bone [9, 10]. If such bone defect cannot be well repaired, it may lead to pathological fracture, which often needs another higher level operation, thus increasing the pain and economic burden of patients.

With the progress of the biomimetic preparation of biomaterials, an artificial biomimetic mineralized collagen (MC) that mimics nanoscale fundamental unit of the natural bone was developed as a novel bone graft substitute. Such material is a biomimetic composite material that simulates the chemical composition and microstructure of natural bone matrix. Relevant animal experiments have shown that the biomimetic MC can effectively guide bone tissue regeneration and repair bone defect. After using in traumatic bone defect and bone fusion after spinal surgery, it has a good performance in guiding bone regeneration [11–14]. However, for the bone defect induced by curettage of benign bone tumors with high risk of recurrence and pathological fracture, clinical observation of the treatment of such bone defect by using biomimetic MC was not reported.

This study retrospectively analyzed 31 patients with benign bone tumors admitted to our hospital from January 2014 to June 2017. The differences between two groups of patients treated with biomimetic MC bone graft or autogenous bone for bone grafting after curettage of benign bone tumors, respectively, were compared through functional and radiological scores after surgery. This retrospective study was approved by Ethics Committee of the Second People’s Hospital of Lianyungang.

## Materials and methods

### General information

According to inclusion and exclusion criteria, a retrospective analysis was performed for 31 patients with benign bone tumors admitted to our department from January 2014 to June 2017. Inclusion criteria: (i) benign bone tumors were diagnosed by X-ray, computerized tomography (CT) or magnetic resonance imaging (MRI) and pathological diagnosis after operation; (ii) informed consent was signed before operation; (iii) effective follow-up could be carried out. Exclusion criteria: (i) postoperative pathological diagnosis of nonbenign bone tumors; (ii) combined with systemic diseases affecting the healing of tumors after surgery: such as metabolic diseases, tumors, malnutrition; (iii) mental disorders, brain diseases, etc. cannot cooperate with late treatment and functional evaluation; (iv) other reasons cannot be effectively followed up. Thirty-one patients include 5 cases of bone cyst, 8 cases of endochondroma, 1 case of fibroxanthoma, 6 cases of nonossifying fibroma, 7 cases of osteoid osteoma and 4 cases of giant cell tumors of bone (2 cases of type I, 2 cases of type II), aged 9–54 years, including 13 males and 18 females, of whom 4 cases were complicated with pathology fracture (2 cases of bone cyst, 1 case of giant cell tumor and 1 case of nonossifying fibroma). Among them, 18 cases used autogenous bone (autogenous bone group) and 13 cases used biomimetic MC (MC group). The detail information of the patients is listed in Table 1.

**Table 1.** Information of the patients

Parameters	Autogenous bone group	MC group
Number of patients	18	13
Mean age (years)	25.94 (9–50)	23.08 (10–54)
Gender (male/female)	8/10	5/8
Cases of diseases		
Bone cyst	3	2
Endochondroma	5	3
Fibroxanthoma	0	1
Nonossifying fibroma	4	2
Osteoid osteoma	3	4
Giant cell tumors of bone	3	1
Average bone defect volume after removal of benign tumor (cm^3^)	4.90 (0.56–13.95)	2.59 (0.72–8.96)
Average bone grafting volume (cm^3^)	5.28 (1–14)	3.23 (2–9)

Although the MC is a commercialized product, the phase composition was also identified by X-ray diffraction (XRD, D/max-2500×) using monochromatic CuK_α_ radiation (*λ* = 0.154 nm, 40 kV, 40 mA). MC was ground into powders and screened by a 200-mesh sieve. XRD data were collected over the 2*θ* range from 10° to 80° with a step size of 0.0197°. The pattern was compared with International Center for Diffraction Data (ICDD) standard PDF cards to identify the phase composition.

### Surgical methods

According to the patient’s condition, appropriate anesthesia method was selected. After the anesthesia came into effect, routine disinfection was performed. Corresponding surgical approaches were adopted according to different parts. After considering the benign tumor in combination with the frozen pathology during the operation, the tumor was completely curetted and the bone defect cavity was burned with electric knife. The bone defect cavity was irrigated by normal saline and was implanted with autogenous bone harvested from iliac crest or MC bone graft substitute (produced by Beijing Allgens Medical Science and Technology Co. Ltd), with compaction bone grafting, to ensure fully filling the bone defect. The selection of bone grafting materials for each patient was determined after pre-operative conversation. Most of the patients with large area of benign bone tumor tended to use autologous bone. In the case of fracture, corresponding internal fixation was applied in the operation. No active bleeding was detected, the incision was closed layer by layer, and drainage tubes or strips were routinely retained in the operation area. All the patients with fracture were treated with braces and other external fixations to protect the limbs.

### Observation indicators

The definite diagnosis of benign bone tumors was confirmed according to the results of postoperative pathological examination, which was performed by Department of Pathology in our hospital and the result would be reported in 2–5 days. In order to evaluate healing results of the surgical site for each patient in the follow-up period, imaging examinations, Lane–Sandhu X-ray scoring and Musculoskeletal Tumor Society (MSTS) scoring were performed 1 month, 6 months and 12 months after operation [15]. As a scoring criterion to evaluate the process of fracture union, Lane–Sandhu X-ray scoring includes bone formation, bone connection and bone remodeling. MSTS is a functional assessment of pain, limb function, patient satisfaction, brace assistance, lower limb (walking, gait) or upper limb (upper limb posture, upper limb lifting ability).

### Statistical analysis

All data were double-checked correctly by two persons, and statistical analysis was carried out by SPSS 22.0. All data were expressed by $\bar{x}$ ± SD. Variables data were tested using *χ*^2^ test, and enumeration data were tested by *t*-test. *P* < 0.05 showed significant statistical difference.

## Results

### Phase identification

**Figure 1.**
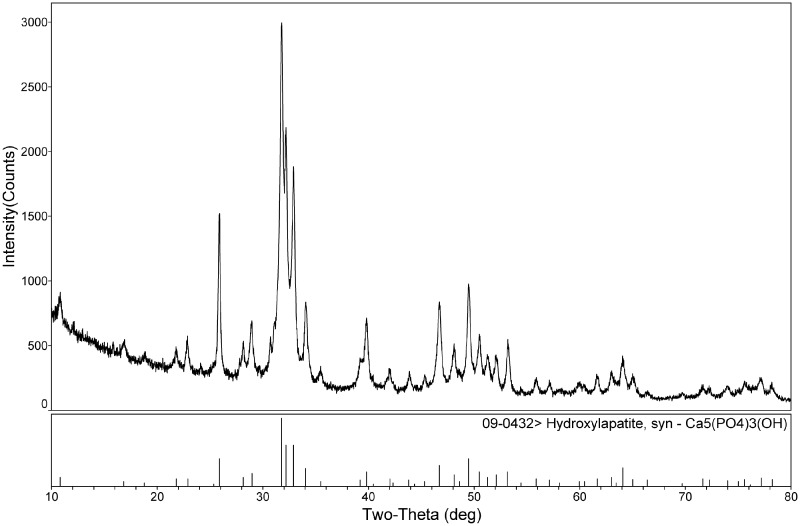
XRD pattern of the commercialized MC bone graft material.

The XRD pattern in Fig. 1 shows the inorganic phase composition of the MC bone graft substitute used in the current study. The diffraction peaks corresponded to characteristic peaks of hydroxyapatite (HA; ICDD PDF #09-0432), and no other peaks were observed. Therefore, the main inorganic phase of the MC was composed of HA, which was in conformity with the major inorganic component of human bone tissue.

### General clinical results

After the operation, wounds of two patients had swelling. After dressing change, the wound healed in the grade order as follows: Grade A, good early stage healing without adverse reactions; Grade B, poor healing with inflammatory responses, such as swelling, callus, hematoma, effusion, but without festering; Grade C, festering at the incision and surgical incision and drainage are needed. One patient had wound exudation, no bacterial growth was observed in bacterial culture. This patient was considered as fat necrosis, and wound healed in Grade A after debridement and dressing change. All the patients in the MC group had no fever, allergy or rejection occurred after the operation.

One case (osteoid osteoma) in autogenous bone group recurred that bone resorption arose in his bone implant area 1 year after the operation. Extended curettage was performed again, and the bone defect was implanted with biomimetic MC and autogenous bone. The bone defect healed well without recurrence again. In such combined use, MC and autogenous bone conducted and induced bone regeneration together. The biomimetic MC was served as scaffold for ingrowth of the osteocytes, while the autogenous bone was able to provide a portion of scaffold as well as useful living osteocytes and growth factors. So, this combination manner minimized the amount of autogenous bone harvesting and decreased related complications at the donor site.

### Follow-up results and statistical results

**Figure 2.**
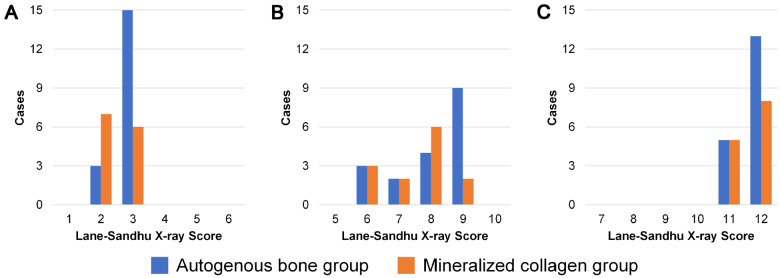
Lane–Sandhu X-ray scores of clinical outcomes in this study at (**A**) 1 month, (**B**) 6 months and (**C**) 12 months after the operation.

All patients were followed up for 9–24 months. Lane–Sandhu X-ray score for each group at 1, 6 and 12 months after the operation are shown in Fig. 2 and statistical data are listed in Table 2. At 12 months after operation, all patients scored more than 10 points, indicated bone healing results were good. Statistical data of MSTS scores are listed in Table 2. MSTS scores of the both groups increased with time going by after the operation, demonstrating activities of daily living of the patients became better and better.

**Table 2.** Lane–Sandhu X-ray scores of the patients

Groups	1 month after operation	6 months after operation	12 months after operation
Autogenous bone	2.83±0.383	8.16±1.162	11.78±0.428
MC	2.46±0.519	7.54±1.050	11.62±0.506
*T*	2.298	1.272	0.966
*P*	0.029	0.217	0.342

Statistical analysis shows that Lane–Sandhu X-ray score of the autogenous bone group was superior to the MC group at 1 month after the operation, but there was no statistical difference between the two groups in the following time (Table 2). The biomimetic MC was superior to the autogenous bone in the functional score at 1 month and 6 months after the operation, and there was no statistical difference between the two groups at 12 months after the operation (Table 3).

**Table 3.** MSTS scores of the patients

Groups	1 month after operation	6 months after operation	12 months after operation
Autogenous bone	17.33±0.383	23.17±1.618	26.78±2.184
MC	21.00±2.000	26.62±2.329	28.02±1.044
*T*	4.686	4.874	2.801
*P*	0.000	0.000	0.179

### A typical case

**Figure 3.**
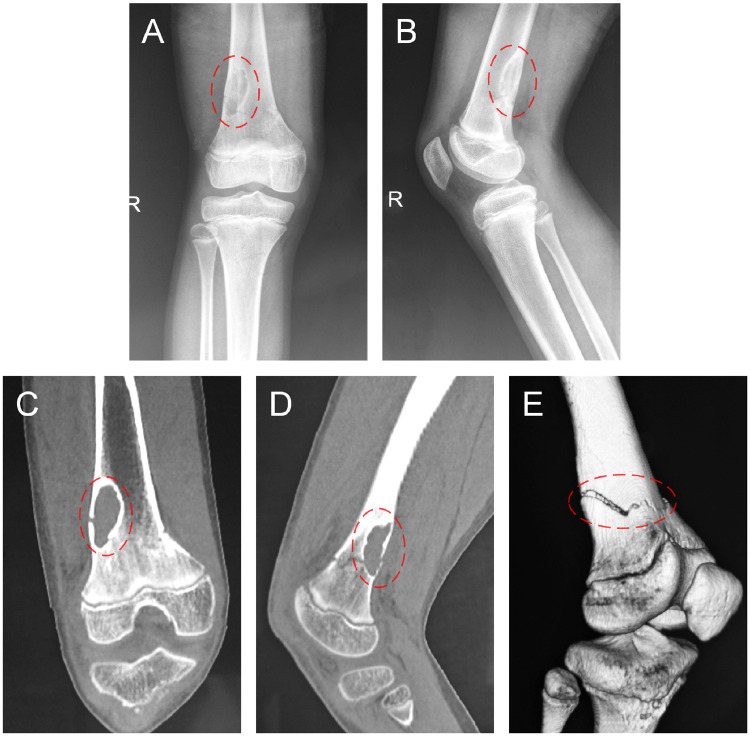
Preoperative imageological examinations of the patient. An oval shadow was located at distal of femur on frontal (**A**) and lateral (**B**) views of plain radiography; evident cavity was shown on coronal (**C**) and sagittal (**D**) CT images; an obvious pathological fracture could be observed on 3D reconstruction of CT (**E**). (the lesion is noted by dashed circle in each picture).

A 13-year-old boy was admitted to the hospital for pain and movement limitation after sprain at a physical education lesson. X-ray and CT examination showed that the distal femoral space-occupying lesion was complicated with pathological fracture (Fig. 3). Open reduction and internal fixation with lesion curettage and bone grafting with biomimetic MC were performed. About 6 g of MC was implanted during the operation. Postoperative pathological diagnosis was bone cyst.

**Figure 4.**
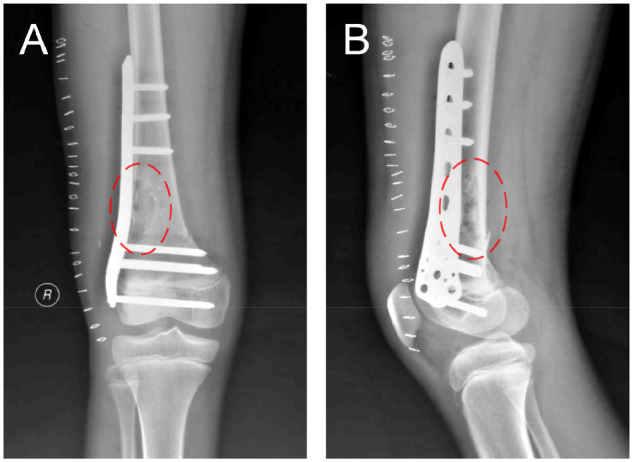
Frontal (**A**) and lateral (**B**) views of plain radiography at 3 days after the operation (the surgical site is noted by dashed circle in each picture).

**Figure 5.**
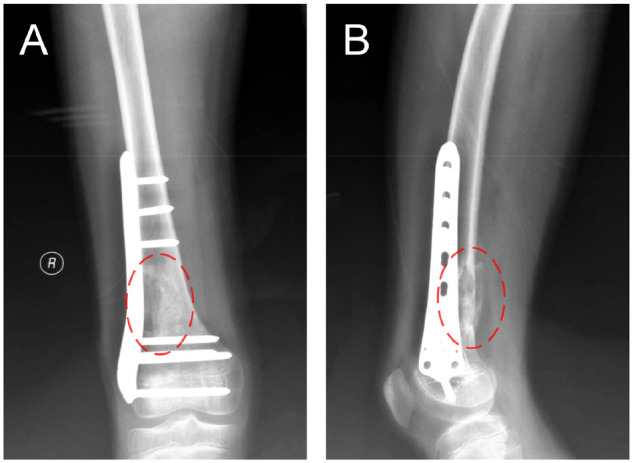
Frontal (**A**) and lateral (**B**) views of plain radiography at 1 month after the operation (the surgical site is noted by dashed circle in each picture).

**Figure 6.**
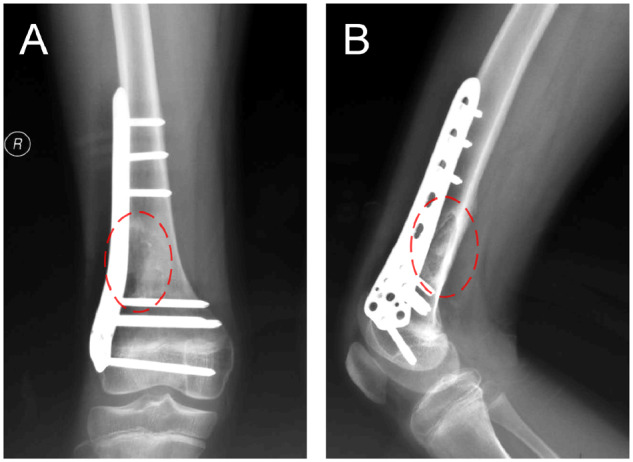
Frontal (**A**) and lateral (**B**) views of plain radiography at 6 months after the operation (the surgical site is noted by dashed circle in each picture).

**Figure 7.**
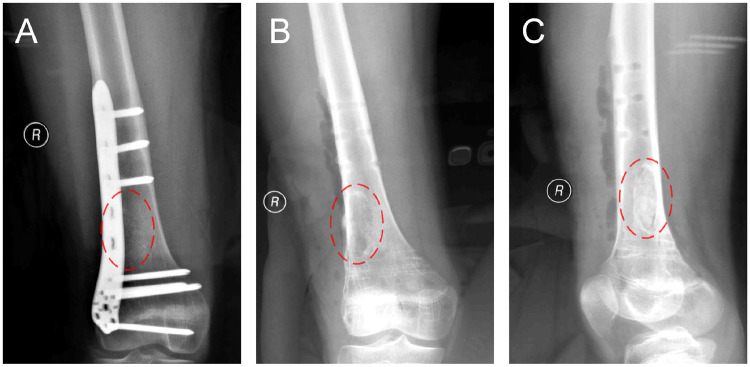
Plain radiography before (**A**) and after (**B**, frontal view; **C**, lateral view) removal of the internal fixation at 9 months after the operation (the surgical site is noted by dashed circle in each picture).

**Figure 8.**
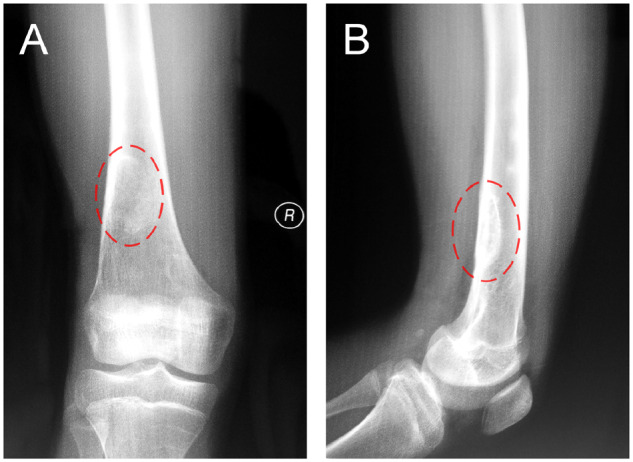
Frontal (**A**) and lateral (**B**) views of plain radiography at 12 months after the operation (the surgical site is noted by dashed circle in each picture).

**Figure 9.**
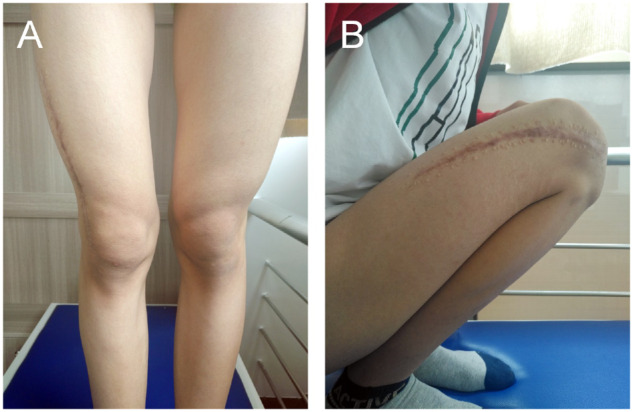
Frontal (**A**) and lateral (**B**) views of the recovered surgical site of the patient at 12 months after the operation.

The patient was examined by X-ray at 3 days (Fig. 4) and 1 month (Fig. 5) after the operation. At 1 month after the operation, Lane–Sandhu X-ray scored 3 points and MSTS scored 14 points. The fracture reduced well with reliable internal fixation, and bone tumor cavity was filled with MC. At 6 months after the operation, Lane–Sandhu X-ray scored 8 points, and MSTS scored 29 points. The frontal position X-ray film (Fig. 6a) shows that the lucent shadow of the cavity decreased, indicating that the trabecula regenerated well; the cortex of the bone was continuous in the lateral position (Fig. 6b), demonstrating the fracture healed; and calcification appeared around the cavity, proving that the bone healed well. At 9 months after operation, the fracture was healed (Fig. 7a). Then the internal plate was removed, the frontal X-ray film (Fig. 7b) shows that the lucent shadow of the cavity disappeared, the trabecula further regenerated; the lateral X-ray film (Fig. 7c) shows calcification appeared in the cavity, proving the new bone formation in cavity. In the final follow-up at 12 months after operation, Lane–Sandhu X-ray scored 11 points, and MSTS scored 30. The frontal X-ray film (Fig. 8a) shows that new bone has well formed, which was similar to normal bone; the lateral X-ray film (Fig. 8b) shows more calcification appeared in the cavity, demonstrating that new bone further regenerated and was waiting for future bone reconstruction. Fig. 9 shows the recovered surgical site of the patient at that time. The well-recovered patient could do normal activities such as walk and squat.

### One case of autogenous bone group

**Figure 10.**
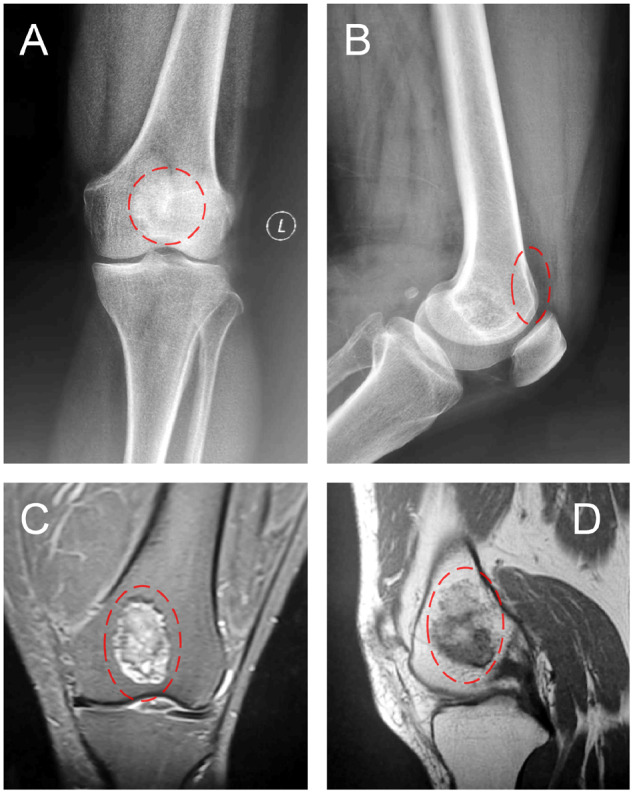
Preoperative imageological examinations of distal femoral space-occupying lesion of the 45-year-old female patient (**A**, frontal view of plain radiography; **B**, lateral view of plain radiography; **C**, coronal MRI; **D**, sagittal MRI; the lesion is noted by dashed circle in each picture).

**Figure 11.**
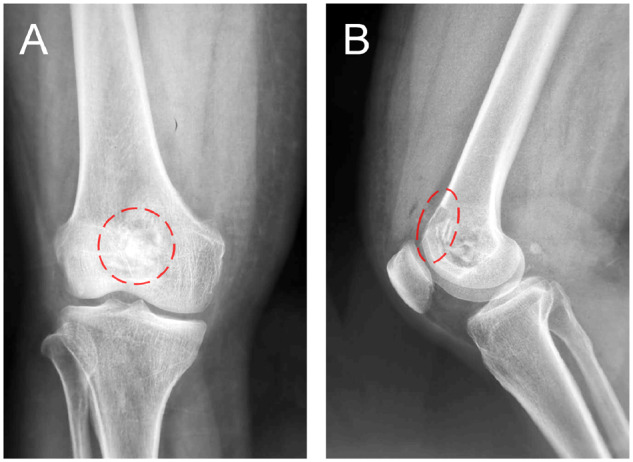
Immediate frontal (**A**) and lateral (**B**) views of plain radiography after the operation (the surgical site is noted by dashed circle in each picture).

**Figure 12.**
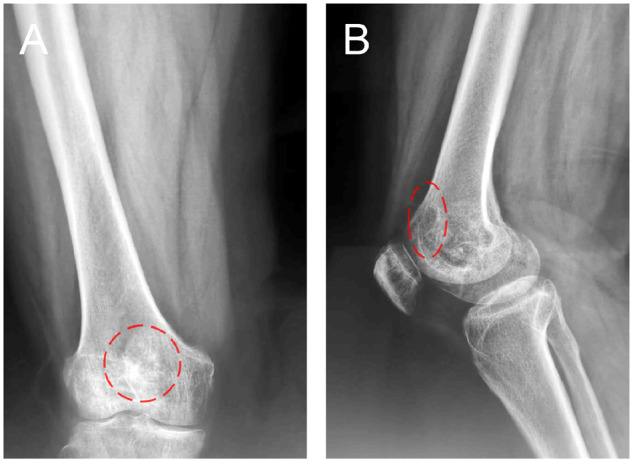
Frontal (**A**) and lateral (**B**) views of plain radiography at 6 months after the operation (the surgical site is noted by dashed circle in each picture).

**Figure 13.**
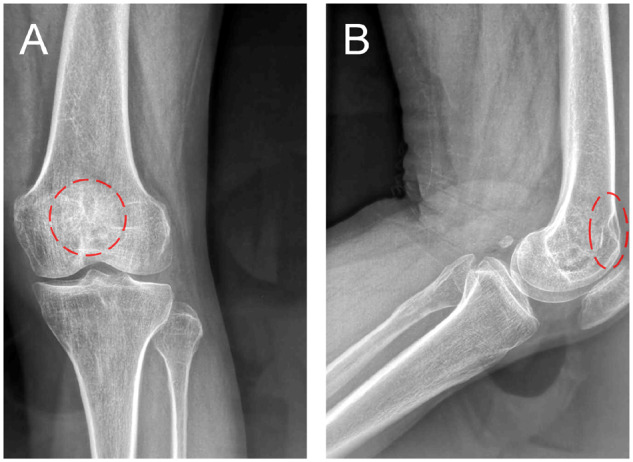
Frontal (**A**) and lateral (**B**) views of plain radiography at 12 months after the operation (the surgical site is noted by dashed circle in each picture).

**Figure 14.**
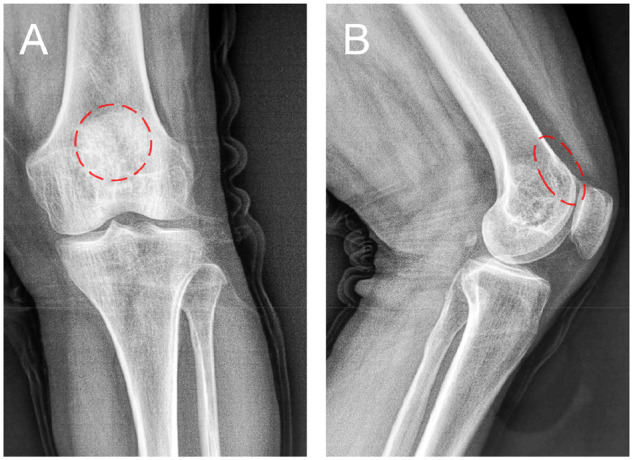
Frontal (**A**) and lateral (**B**) views of plain radiography at 24 months after the operation (the surgical site is noted by dashed circle in each picture).

A 45-year-old woman was admitted to the hospital for pain at her left knee joint for 1 year. X-ray examination showed distal femoral space-occupying lesion (Fig. 10a and b), and MRI showed a benign bone tumor (Fig. 10c and d). A selective operation was performed with lesion curettage and bone grafting using ipsilateral autogenous iliac bone. Postoperative pathological examination confirmed the benign bone tumor was nonossifying fibroma. X-ray examination showed the tumor was completely curetted, and the defect was fully filled with the autogenous bone (Fig. 11). Lane–Sandhu X-ray scored 3, 9 and 12 points, and MSTS scored 14, 20 and 24 points at 1-month, 6-month (Fig. 12) and 12-month (Fig. 13) follow-ups, respectively. At 2 years after the operation, the new bone has well formed and was indiscernible from surrounding normal bone tissues (Fig. 14). The patient recovered very well.

## Discussion

The accurate incidence of benign bone tumors cannot be measured because most of them are asymptomatic, but it is undeniable that benign bone tumors are common diseases in clinic, including: nonossifying fibroma, giant cell tumors of bone, endochondroma, bone cysts, etc. [16]. In this study, only 14 patients had pain symptoms, among them 4 patients had pathological fractures. Most of the benign bone tumors had no clinical manifestations throughout patient’s life. Although there is no consensus on the treatment of benign bone tumors, at present, many methods have been being developed for the treatment of benign bone tumors, such as surgical resection, percutaneous injection of sclerosing agents or corticosteroids, radiotherapy, etc. [17]. With the establishment of Enneking’s surgical staging system for musculoskeletal system tumors, intracapsular resection of benign bone tumors and tumor-like diseases is the major surgical treatment method [18]. For the treatment of cysts after resection of benign bone tumors, most scholars believe that the cavity after the resection needs to be filled with bone grafting materials to reconstruct the bone structure and restore the mechanical strength of bone [16–19].

At present, the commonly used bone grafting materials in clinic are autogenous bone, allogeneic bone, bone cement, many kinds of artificial bone grafts, etc. Autogenous bone has advantages of rapid osteogenesis, no special storage, no immune response, good bone conduction, osteoinduction and osteogenic cells. Autogenous bone is considered as the “gold standard” for bone grafting [4–7]. Children and adolescents are the high incidence age group of benign bone tumors [20]. However, the donor sites of children and adolescents with benign bone tumors are very limited, excessive bone removal can lead to fracture of the donor site [21]. Besides common donor site complications, bone extraction of children and adolescents may damage epiphysis, thereby affecting physiological growth and development of the bone [22]. Adding new surgical sites, prolonging operation time, increasing bleeding, increasing operation risk and complications of the donor site are also the shortcomings of the autogenous bone. Autogenous bone cannot provide specific structure, thereby limiting its clinical use in special curettage. In this study, the MSTS score of autogenous bone group was lower than that of MC group at 1 month after the operation. The pain of donor site was often greater than that of tumor curettage, although it does not exclude the influence of psychological factors. However, as the attachment site of abdominal muscle, the pain of iliac bone was more obvious after the operation, such complication affected the functional abilities of patients. With the progress of bone repair, there was no significant difference between the two groups at 12 months after operation. For the adolescents with high incidence of benign bone tumor, whether iliac extraction affects subsequent growth and development of the donor site bone remains to debate.

In view of the above shortcomings, efforts have been made to find a new type of bone graft substitute. Allogenic bone, xenogeneic bone, a variety of metals, ceramics, polymers and their composites have been developed as bone substitute materials [23–25], but these are still not comparable to autogenous bone [4–7]. With the thorough and meticulous study of the natural bone, hierarchical structure of bone and the formation mechanism of the same were scientifically and accurately demonstrated. The natural MC mainly made up of orderly arranged nano-sized HA and type I collagen is considered as the fundamental structural unit of the bone extracellular matrix [26–28]. Based on the characteristics of unique collagen/HA composition and hierarchical structure of the natural bone, biomimetic artificial MC composed of type-I collagen and nano-sized HA has been designed and prepared via an *in vitro* biomimetic mineralization process. Within the biomimetic MC, HA is a poorly crystallized nanoparticle, which is arranged periodically and orderly among the collagen molecules. The biomimetic MC possesses the same chemical composition and microstructure as the natural bone matrix of human body, which can provide a good microenvironment for the activity of bone cells and help guide the regeneration of bone tissue [11–14]. In this study, although the MSTS score of the MC group was initially better than that of the autogenous bone group, the Lane–Sandhu X-ray score of the MC group was significantly lower in imaging with statistically significant difference at 1 month after the operation. The main reason was that there were osteoblasts and growth factors in autogenous bone, while MC bone grafting material needed the ingrowth of the osteoblasts, as well as delivery of growth factors, then the material would be creepingly substituted by surrounding bone tissue to form new bone. However, the Lane–Sandhu X-ray score did not show statistically significant difference at 6 and 12 months after operation, indicating that the biomimetic MC biomaterial has good osteoconduction and bone regeneration performance. Recent basic studies also showed that the biomimetic MC can effectively promote and induce bone marrow mesenchymal stem cells to differentiate into osteoblasts, so as to promote fracture healing [29, 30]. This also explains the observation results that the two groups in this study did not have statistics differences. As an artificial biomimetic biomaterial, MC can also reduce immune rejection, avoid donor complications, has simple implantation operation and appropriate plasticity. Although there were three cases of wound swelling or exudation in the MC group, they were considered as fat necrosis and normal inflammation of wound rather than rejection reaction.

In previous studies, MC is a biodegradable material that could be substituted by regenerated bone tissue. In this study, due to the low density of the porous MC, the bone grafts cannot be directly observed on imaging examination in follow-ups. However, as time goes on, the shadow on the curettage site gradually disappeared and was occupied by the regenerated radiopaque bone tissues. For those cases in the MC group with internal fixation, the bone grafting material cannot be found during the surgery removing the internal fixation. With reference to previous animal experiments, the MC should be fully biodegraded and substituted by regenerated bone.

In this study, recurrence of benign bone tumor was not observed in the implantation sites of the MC. It has been reported that nano-sized HA or composites containing nano-sized HA have a certain inhibitory effect on bone tumor [31–33]. The HA within the MC is also in the form of nano-sized particles, which may play a role in inhibiting tumor recurrence. The specific mechanism of recurrence free needs further theoretical and practical studies. The present study does not exclude the influence of sample selectivity error and confounding factors, which needs further study with larger sample size and discussion.

## Conclusion

To sum up, MC can be used as the bone graft substitute material for bone defect repair after curettage of benign bone tumors. MC performed not as good as autogenous bone in early stage of bone healing but achieved comparable outcomes as autogenous bone after long-term follow-ups. At the same time, MC has advantages in function recovery after the operation as well as avoided potential complications induced by harvesting autogenous bone. As a result, the artificial biomimetic MC can be used as a good autogenous bone substitute material for the treatment of benign bone tumors.
